# Associations of thalamocortical networks with reduced mindfulness in alcohol use disorder

**DOI:** 10.3389/fpsyt.2023.1123204

**Published:** 2023-07-06

**Authors:** Niklaus Denier, Leila M. Soravia, Franz Moggi, Maria Stein, Matthias Grieder, Andrea Federspiel, Zeno Kupper, Roland Wiest, Tobias Bracht

**Affiliations:** ^1^Translational Research Center, University Hospital of Psychiatry and Psychotherapy, University of Bern, Bern, Switzerland; ^2^Translational Imaging Center (TIC), Swiss Institute for Translational and Entrepreneurial Medicine, Bern, Switzerland; ^3^Clinic Suedhang, Kirchlindach, Switzerland; ^4^Department of Clinical Psychology and Psychotherapy, Institute of Psychology, University of Bern, Bern, Switzerland; ^5^Institute of Diagnostic and Interventional Neuroradiology, University of Bern, Bern, Switzerland

**Keywords:** magnetic resonance imaging, alcohol use disorder, mindfulness, thalamus, default mode network, salience network

## Abstract

**Background:**

Increased mindfulness is associated with reduced alcohol consumption in patients with alcohol use disorder (AUD) after residential treatment. However, the underlying neurobiological mechanism of mindfulness in AUD is unclear. Therefore, we investigate the structural and functional alterations of the thalamocortical system with a focus on the mediodorsal thalamic nucleus (MD-TN), the default mode and the salience network (DMN/SN) which has previously been associated with mindfulness in healthy subjects. We hypothesized lower mindfulness and reduced structural and functional connectivity (FC) of the thalamocortical system, particularly in the DMN/SN in AUD. We assumed that identified neurobiological alterations in AUD are associated with impairments of mindfulness.

**Methods:**

Forty-five abstinent patients with AUD during residential treatment and 20 healthy controls (HC) were recruited. Structural and resting-state functional MRI-scans were acquired. We analysed levels of mindfulness, thalamic volumes and network centrality degree of the MD-TN using multivariate statistics. Using seed-based whole brain analyses we investigated functional connectivity (FC) of the MD-TN. We performed exploratory correlational analyses of structural and functional DMN/SN measurements with levels of mindfulness.

**Results:**

In AUD we found significantly lower levels of mindfulness, lower bilateral thalamic and left MD-TN volumes, reduced FC between MD-TN and anterior cingulum/insula and lower network centrality degree of the left MD-TN as compared to HC. In AUD, lower mindfulness was associated with various reductions of structural and functional aspects of the MD-TN.

**Conclusion:**

Our results suggest that structural and functional alterations of a network including the MD-TN and the DMN/SN underlies disturbed mindfulness in AUD.

## 1. Introduction

Alcohol use disorder (AUD) is a chronic disorder and leads to severe physical, psychological, and social consequences (1). Furthermore, AUD is characterized by high relapse rates (2). Therefore, alcohol related direct and indirect costs are immense on an individual as well as on a societal level (3). Despite significant progress in the development of efficacious psychological and pharmacological treatments for AUD, one-year relapse rates remain with more than 50% very high (4–6), highlighting the need to improve treatment regimens and to identify factors that contribute to better treatment outcomes. The relapsing nature of substance addiction has made it clear that relapse prevention is an essential treatment component for long-term recovery, shifting attention to interventions that propose to address this need such as mindfulness-based relapse prevention (MBRP). A recent review could show that despite some heterogeneity regarding the type of MBRP program used, results support the effectiveness of these interventions in substance use, especially in reducing craving, decreasing the frequency of substance use, and improving depressive symptoms (7).

Acute and chronic effects of alcohol consumption are known to alter the state of consciousness by propagating mind-wandering, characterized by undirected stream of thoughts (8), reduced meta-consciousness (9), weakened overall self-control (10) and increased impulsivity (11). Mindfulness, a mental state opposite to mind-wandering (12, 13), is defined by the awareness that arises from paying non-judgmental attention in the experience of the present moment (14). Mindfulness can be construed as a stable psychological trait and quantified with questionnaire-based inventories demonstrating good test-retest reliability (15, 16). Trait mindfulness refers to an individual’s inherent capacity to be aware and attentive to the present moment, both internally and externally, across various situations. However, research has specified that mindfulness nonetheless may change over longer timeframes, e.g., due to mindfulness practice, mindfulness based psychotherapeutic interventions and other factors (16, 17). Generally, mindfulness has a positive impact on mental health (18) and is positively associated with engagement in various health behaviours (19). In patients with AUD, higher levels of mindfulness are known to be associated with reduced alcohol consumption (20), with lower craving (21) and reduced impulsivity traits in AUD (11, 22). Improvement of mindfulness through specific mindfulness based treatment programs, such as mindfulness-based relapse prevention, offer a possibility to improve alcohol-related outcomes, including abstinence or drinking goals, well-being and life satisfaction, in AUD (23–25).

On a neurobiological level, mindfulness is associated with activation shifts within the default mode (DMN) and salience network (SN) (26–28). The posterior cingulate cortex (PCC) and precuneus are core integrative hubs of the DMN and increased glucose metabolism and functional integration of those regions are linked to high levels of mindfulness (29, 30). High levels of mindfulness are also linked to lower thalamic glucose metabolism (29), while meditation is associated with higher thalamic glucose metabolism than a restful state (31). The thalamus shows higher functional integration within the SN, including the anterior cingulate cortex (ACC) and insula in subjects with high levels of mindfulness (32). The thalamus and especially the mediodorsal thalamic nucleus (MD-TN) play a core role in orchestrating the dynamic selection of cortical representations (33) and have been proposed to be linked with impaired response inhibition and excessive salience attribution to alcohol-related cues in AUD (34). Through its reciprocal connections with cortical regions including the DMN and SN, the MD-TN integrates and amplifies mental representations (35–39) relevant to the attribution of emotional valence (40, 41), which translates into habitual behaviour (35, 38). Given that AUD has also been conceptualized as a disorder which is characterized by automatized, habitual behaviours dominating over goal-directed actions (42, 43) the investigation of MD-TN, its connections and its relation to mindfulness in AUD is of high clinical importance.

This is the first study to investigate the role of the thalamocortical system for mindfulness in patients with AUD. It is the aim of this study to compare structural and functional properties of the thalamocortical system between a group of recently abstinent patients with AUD and healthy controls (HC). In addition to structural MRI analyses, we investigate seed-based resting-state functional connectivity (FC) (44, 45) of the MD-TN. This analysis is complemented by a graph theory approach allowing conclusions on functional integration of the MD-TN within overall functional networks. We are interested in the specific role of the MD-TN and its connectivity with the DMN and SN. We expect (1) reduced levels of mindfulness in AUD compared to HC and (2) alterations of the thalamocortical system in AUD compared to HC. Specifically, we hypothesize reduced thalamic and MD-TN volumes and decreased functional integration and communication of the MD-TN with functional networks (DMN, SN) in AUD compared to HC. (3) Associations of reduced structural and functional properties of the thalamocortical system in AUD with levels of mindfulness.

## 2. Materials and methods

### 2.1. Study participants

Participants are a subsample of a clinical trial investigating the effects of an alcohol-inhibition training on relapse and drinking behaviour after treatment in patients with AUD (clinicaltrials.gov: NCT02968537; Swiss National Clinical Trials Portal: SNCTP000002043) (46, 47) and includes subjects of previous cross-sectional neuroimaging studies (48, 49). We included 46, right-handed patients with AUD attending an 8 or 12 weeks abstinence-oriented residential treatment program at a specialized treatment center for addiction in Switzerland (Clinic Suedhang). Diagnosis of AUD were assessed in accordance with DSM-5 criteria. Other severe substance use disorder (except nicotine; drug use identification test; DUDIT ≥25 per substance (50)) and diagnosed neurocognitive problems (e.g., Korsakoff syndrome) were exclusion criteria. We also assessed total number of alcohol detoxifications in the past and days of abstinence since MRI measurement. Twenty right-handed healthy controls (HC) counterbalanced for age and sex were included for comparative analyses. Inclusion criterion was non-problematic drinking behaviour, as assessed with the alcohol use disorders identification test (AUDIT, score < 8), alcohol use disorder scale (AUD-S 49, score <2), and low scores regarding psychopathology [brief symptom check list (BSCL 50, GSI *t*-value ≤63)]. For more details of the main study, see Tschuemperlin et al. (47).

The study and experimental protocols were approved by the cantonal ethics committee of Bern (KEK Bern; KEK-number: 2016-00988). All participants provided written informed consent after a full explanation of the procedures involved and were reimbursed with 50 Swiss Francs for participation. The study was performed in accordance with relevant regulations and guidelines.

### 2.2. Assessment of mindfulness

Mindfulness was assessed with the comprehensive inventory of mindfulness experience (CHIME) (15, 16). The CHIME consists of 37 questions with a five-point rating-scale (0: never true; 4: always true), that are assigned to eight different factors of mindfulness including (1) inner awareness (awareness towards internal experiences), (2) outer awareness (awareness towards external experiences), (3) acting with awareness, (4) acceptance (accepting and non-judgmental orientation), (5) decentering (decentering and non-reactivity), (6) openness (openness to experiences), (7) relativity (relativity of thoughts), (8) insight (insightful understanding) and a total CHIME score (calculated as the mean score of the 8 CHIME subscores) that reflects overall level of mindfulness. Overall, the CHIME shows good internal consistency as indicated by a Cronbach’s alpha of *α* = 0.84 (15, 16).

### 2.3. Multimodal MRI data acquisition

Image acquisition was performed with a 3-T scanner (MAGNETOM Prisma, Siemens Healthineers, Erlangen, Germany) and a 64-channel head/neck coil at the University Hospital of Bern. We used a bias-field corrected MP2RAGE sequence for acquisition of high-resolution T1-weighted images with optimized subcortical contrast. MP2RAGE acquisition parameters were voxel dimension = 1 × 1 × 1 mm^3^, number of slices = 256, matrix size = 256 × 256, field of view = 256 × 256, TE = 2.93 ms, TI = 700 ms, TR = 5,000 ms. Two gradient echo images (INV1 and INV2) and a T1-weighted image (UNI) were generated. For acquisition of a resting-state fMRI with a duration of 6 min 30 s long, we used whole brain echo planar imaging (EPI). Acquisition parameters were volumes = 300, voxel dimension = 2.2 × 2.2 × 2.2 mm^3^, number of slices = 60, matrix size = 104 × 104, field of view = 230 × 230, TE = 37 ms, TR = 1,300 ms. All participants were instructed to close their eyes and to not ruminate.

### 2.4. Volumetry and cortical thickness measurements

To compute basic MRI metrics including intracranial volume (ICV) and brain volume we used *SPM12* (6906) and *MATLAB* R2021a. MP2RAGE images were segmented using *SPM12* in grey matter (GM), white matter (WM) and cerebrospinal fluid (CSF). Volumes were calculated by combing tissue volume: ICV = GM + WM + CSV, brain = GM + WM. Further, we used *HD-BET*, a high-quality deep learning based brain extraction algorithm, with MP2RAGE INV2 images and applied the derived binary mask to the UNI images (51). Next we segmented brain extracted UNI images using *DL + DiReCT*, a deep learning-based neuroanatomy segmentation, followed by a diffeomorphic registration-based cortical thickness (DiReCT) estimation (52). Then, we computed bilateral thalamic volumes and mean cortical thickness. Volumes of the two MD-TN were approximated by transforming a MD-TN template in MNI space to native space using the individually determined inverse normalisation matrix in *SPM12*. We used MD-TN template (MNI space) from the high-resolution DISTAL (DBS Intrinsic Template AtLas, label 37, Nucleus medialis) atlas (53). Resulting native space MD-TN images were binarized using a threshold of >0.5 and approximative volumetry was performed in *MATLAB*.

### 2.5. Functional connectivity and integration of MD-TN

We analysed seed-based whole brain FC using the *CONN 20b* toolbox (54). Native EPI volumes were realigned, and a field map correction was applied. Scrubbing of outlier scans was performed using the artefact detection tools (ART) toolbox implemented in *CONN*. Outliers of the time series were defined as those time points higher than the 98th percentile in in framewise displacement (FD) or global BOLD signal. Additionally, we computed mean FD of motion parameters and mean DVARS, which is the spatial root mean square of the BOLD signal after temporal differencing (55) for every subject. We excluded one subject with a mean FD of 0.36 and a mean DVARS of 12.68. After exclusion there was a still a significant group difference in mean FD (AUD: 0.27 ± 0.08; HC: 0.23 ± 0.06; *T* = 2.16, *p* = 0.035) but not in mean DVARS (AUD: 3.17 ± 1.23; HC: 3.19 ± 0.88; *T* = −0.04, *p* = 0.969). Realigned and scrubbed EPI volumes were co-registered to MP2RAGE UNI volumes, and both normalized to MNI space. Normalized UNI volumes were segmented into GM, WM, and CSF. To remove physiological noise and isolate low frequency fluctuations, we applied a band-pass filter of 0.008–0.09 Hz to all normalized voxel time series. We regressed nuisance variables including 12 realignment parameters and each 5 time series within normalized CSF and WM regions, derived by principal component analysis. To analyse functional integration and connectivity of the MD-TN, we derived a bilateral MD-TN mask from the high-resolution DISTAL (DBS Intrinsic Template AtLas, label 37, Nucleus medialis) atlas (53). Spatial maps of whole brain seed-based FC of the bilateral MD-TN seed were computed by Pearson correlation coefficients of the BOLD time series. To further explore FC of the MD-TN seed to the DMN and SN, we extracted values of *a priori* defined regions including the precuneus, PCC and ACC using masks of the automatic anatomical labelling (AAL) atlas (56).

To analyse network centrality, we computed a graph adjacency matrix ( $A_{ij}$ ) for each subject by thresholding to the correlation coefficient of the bilateral MD-TN to each set of classical functional networks by *z* > 0.5. The networks were provided by the *CONN 20b* toolbox and derived from the human connectome project (57). Then left and right MD-TN’s network centrality degree ( $d_{i})$ as measurement of functional integration, was computed by $d_{i}=\underset{j}{\sum }A_{ij}$ .

### 2.6. Statistical analyses

The Statistical Package for Social Sciences *SPSS 27.0* (SPSS, Inc., Chicago, Illinois) was used for data analyses, where not mentioned otherwise. Demographics and basic brain metrics (ICV, brain volume) between AUD patients and HC were compared using independent *t*-tests, Mann–Whitney *U* tests or *χ*^2^ tests as appropriate for continuous and dichotomous data. Group differences between AUD and HC in levels of mindfulness (CHIME total) and aspects of mindfulness (8 CHIME subscores) were assessed with a MANCOVA controlling for age and gender.

A mixed-model MANCOVA controlling for age, gender, brain volume and years of education was performed with the between-subject factor group (AUD patients, HC), the within-factor hemisphere (left vs. right) and the dependent variables (mean cortical thickness, volume thalamus and volume MD-TN). In case of a significant main group effect, post-hoc ANCOVAs controlling for age, gender, years of education and brain volume were calculated for each volumetric variable. When there was a significant group × hemisphere interaction, we tested each hemisphere separately, otherwise we used a mean score of both hemispheres.

We used the CONN toolbox to compare whole-brain FC maps of the MD-TN between AUD and HC and used age, gender, and mean FD as covariates. We applied a voxel-threshold of *p* < 0.01 and a family wise error (FWE) correction of *p* < 0.05. The network centrality degree of the left and right MD-TN was compared between AUD and HC using two ANCOVAs controlling for age, gender, and mean FD. Level of significance was considered as *α* = 0.05/2 = 0.025, by applying a Bonferroni correction.

### 2.7. Exploratory correlations

To investigate possible association of years of education, employment, and relationship on the level of mindfulness, we performed Spearman correlations for the whole group, as well as for HC and AUD separately. To assess associations between level of mindfulness (CHIME total) and bilateral thalamus volumes, MD-TN volumes and extracted FC values (precuneus, PCC and ACC) we performed Spearman correlation analyses within AUD and the HC group separately. Additionally, we investigated within AUD associations between CHIME subscores as well as AUD related scores (days of abstinence, number of detoxifications before treatment entry) with structural and functional aspects of the thalamocortical system (volume MD-TN, MD-TN-ACC FC, MD-TN-PCC FC, MD-TN-precuneus FC, and MD-TN network centrality degree) using Spearman correlations. Results were visualized by color-coded heat maps.

## 3. Results

### 3.1. Demographics, alcohol related scores and basic brain metrics

AUD patients and HC did not differ regarding age and gender. Patients were significantly less likely to be in a relationship and had fewer years of education. At the time of the MRI assessment, patients were abstinent from alcohol for 32 ± 15 days on average, had an average of three detoxifications and an average of 12 ± 9 years of problematic drinking. The average AUDIT score for patients was 25 (SD = 7) and 4 (SD = 2) for HC. Groups did not differ significantly for ICV, but there was a non-significant trend in reduced brain volume (see Table 1).

**Table 1:** Demographics, basic MRI metrics and alcohol related variables.

	AUD patients (*N* = 45)	Healthy controls (*N* = 20)	*p*-values
*Demography*			
Females/males	17/28	7/13	0.830
Age, years	43.44 (9.27)	40.65 (12.53)	0.189
Relationship (yes/no)	23/22	17/3	0.010^**^
Years of education	14.22 (3.52)	16.42 (3.39)	0.018^*^
Employment (yes/no)	29/16	19/1	0.010^**^
*Basic brain metrics*			
ICV, litre	1.46 (0.12)	1.49 (0.13)	0.478
Brain volume, litre	1.17 (0.10)	1.22 (0.12)	0.068
*Alcohol*			
AUDIT	24.78 (7.11)	3.85 (2.01)	<0.001^***^
Nr. of detox	3.09 (2.86)		
Days of abstinence	32.12 (14.83)		
Years of probl. Drinking	12.20 (9.74)		

ICV, intracranial volume; Nr. detox, number of previous detoxifications; years of probl. drinking, years of problematic drinking; AUDIT, alcohol use disorders identification test. ^*^p < 0.05, ^**^p < 0.01, ^***^p ≤ 0.001.

### 3.2. Group differences in levels of mindfulness

CHIME total was significantly lower in patients with AUD than in HC [3.99 ± 0.58 vs. 4.38 ± 0.47, *F*(1, 61) = 9.66, *p* = 0.003], large effect size *η*^2^ = 0.14. CHIME subscores differed significantly regarding acceptance, decentering and openness (see Table 2).

**Table 2:** Group differences in levels of mindfulness between patients with AUD and healthy controls.

	AUD patients (*N* = 45)	Healthy controls (*N* = 20)	*p*-values	*F*_1,61_ values	Effect size *η*^2^
CHIME total (level of mindfulness)	3.99 (0.58)	4.38 (0.47)	0.003^*^	9.66	0.137
CHIME: inner awareness	4.56 (1.09)	4.88 (0.67)	0.181	1.83	0.029
CHIME: outer awareness	4.77 (0.96)	4.94 (0.88)	0.299	1.10	0.018
CHIME: acting with awareness	4.35 (0.87)	4.32 (0.80)	0.940	0.01	<0.001
CHIME: acceptance	3.26 (1.01)	4.06 (0.98)	0.002^*^	10.92	0.152
CHIME: decentering	3.64 (0.98)	4.32 (0.63)	0.003^*^	9.39	0.133
CHIME: openness	3.24 (0.96)	4.12 (0.81)	<0.001^*^	12.78	0.173
CHIME: relativity	4.06 (0.81)	4.12 (0.98)	0.628	0.37	0.004
CHIME: insight	4.00 (0.72)	4.26 (0.82)	0.150	2.12	0.034

CHIME, comprehensive inventory of mindfulness experience. ^*^p < *α* = 0.05/9 = 0.006.

### 3.3. Group differences in volumes of thalamus, MD-TN and mean cortical thickness

**Figure 1:**
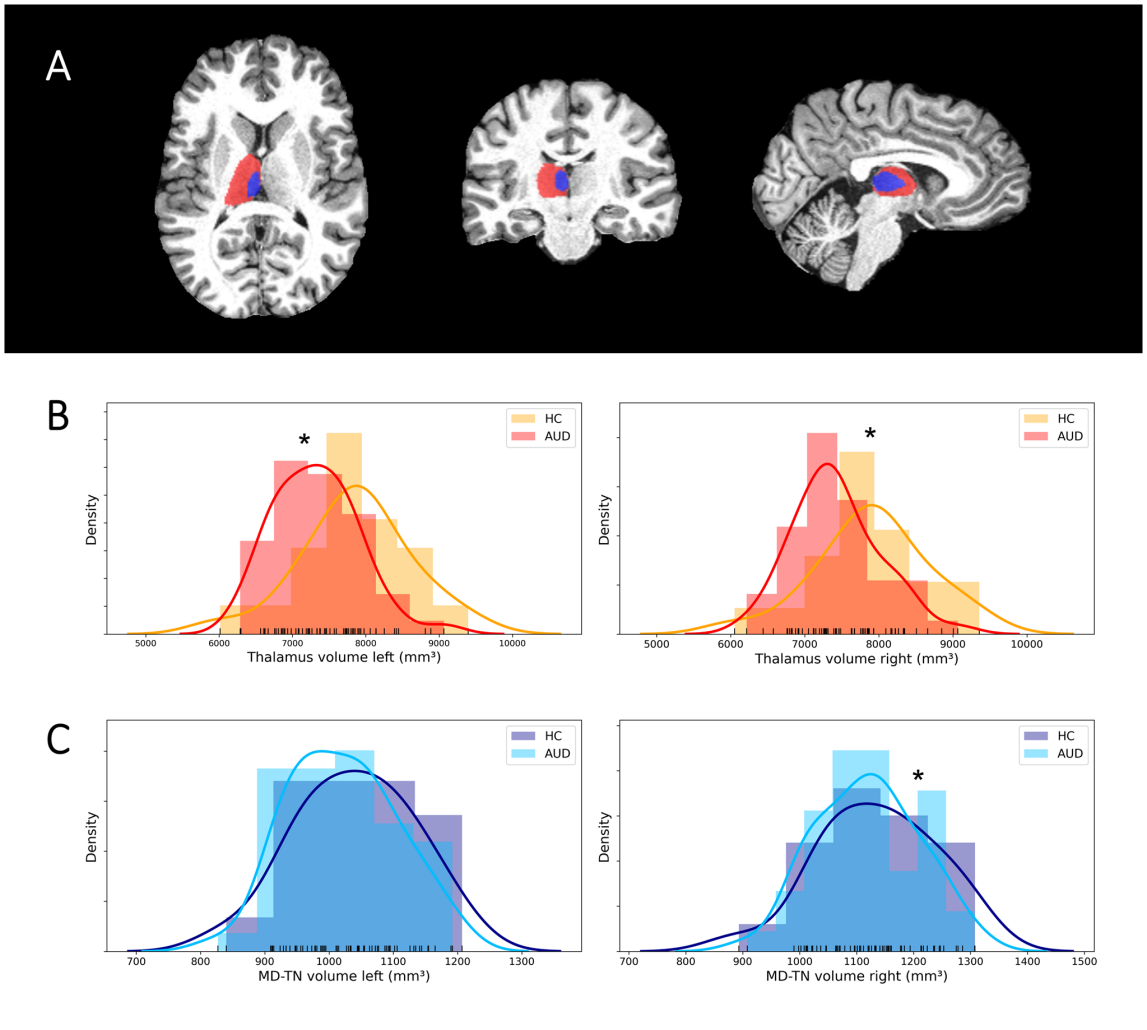
Segmentation of the thalamus and MD-TN and volumetric group differences. **(A)** Sample segmentation of the thalamus (red) and MD-TN (blue) in native space. **(B)** Histograms of group distributions of thalamus volumes. **(C)** Histograms of group distributions of MD-TN volumes. *Significant post-hoc ANVOCA *p*-value.

Comparing patients and HC regarding thalamic volumes and cortical thickness the mixed model MANCOVA revealed a significant main group effect [*F*(1, 58) = 6.18, *p* = 0.016, medium effect size *η*^2^ = 0.096] and a significant hemisphere × group interaction [*F*(1, 58) = 4.90, *p* = 0.031, medium effect size *η*^2^ = 0.078]. Post-hoc tests revealed significantly reduced volumes in patients as compared to HC in the left thalamus [7,315 ± 578 vs. 7,864 ± 779 mm^3^, *F*(1, 58) = 8.69, *p* = 0.005, medium effect size *η*^2^ = 0.130], the right thalamus [7,421 ± 584 vs. 7,907 ± 786 mm^3^, *F*(1, 58) = 5.41, *p* = 0.024, medium effect size *η*^2^ = 0.085] and the right MD-TN [1,119 ± 89 vs. 1,135 ± 107 mm^3^, *F*(1, 58) = 4.221, *p* = 0.044, medium effect size *η*^2^ = 0.068]. There was a non-significant trend for lower volumes of the left MD-TN in AUD [1,021 ± 84 vs. 1,038 ± 95 mm^3^, *F*(1, 59) = 2.96, *p* = 0.090]. There was no significant difference in mean cortical thickness between patients and HC in the left [2.10 ± 0.09 vs. 2.10 ± 0.08 mm, *F*(1, 58) = 0.012, *p* = 0.913] and right hemisphere [2.12 ± 0.09 vs. 2.13 ± 0.09 mm, *F*(1, 58) = 0.225, *p* = 0.637] (see Figure 1).

### 3.4. Group differences in functional connectivity and integration of MD-TN

**Figure 2:**
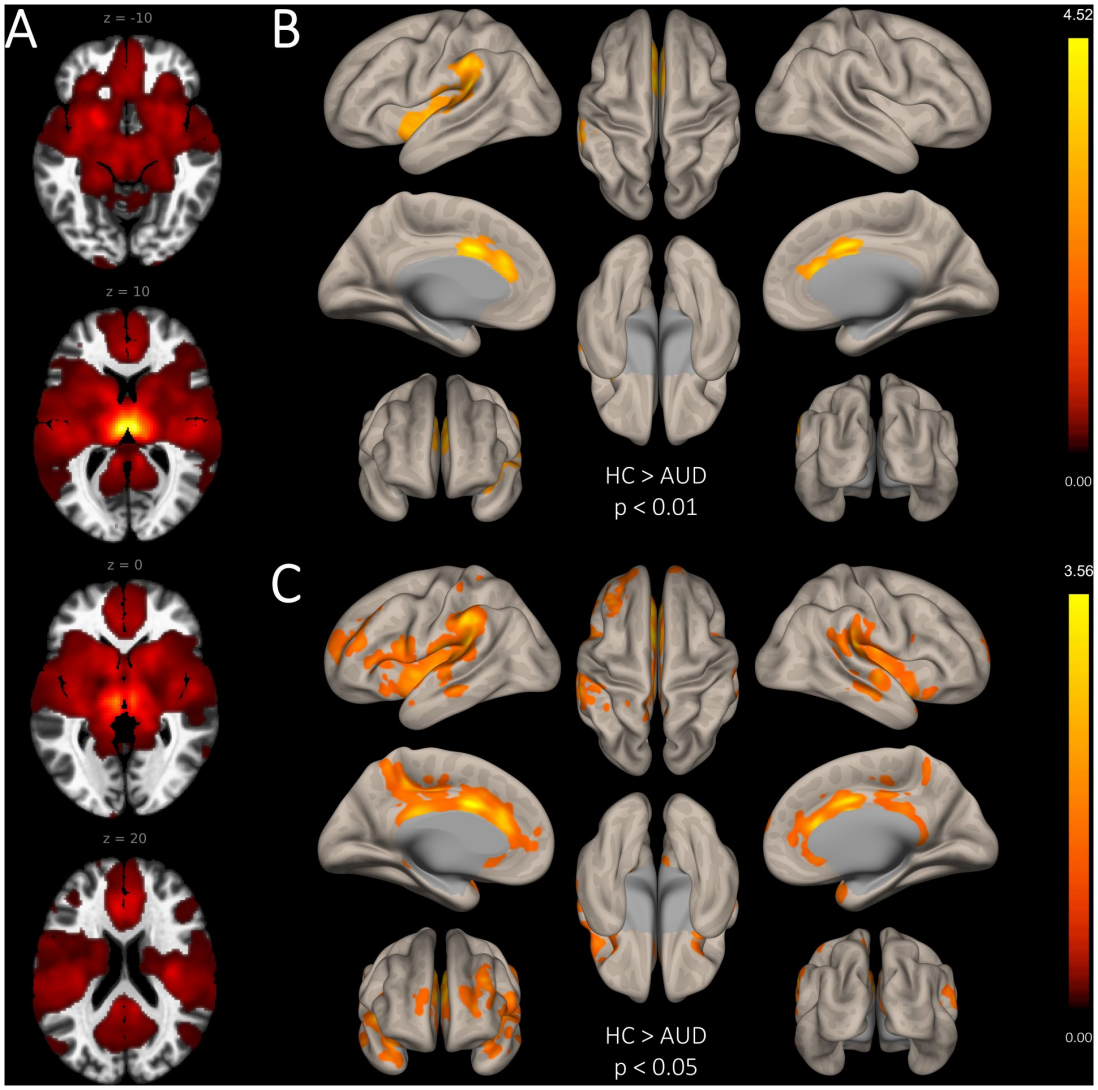
Group differences in seed-based functional connectivity of the bilateral MD-TN. **(A)**. Whole-brain FC map of the bilateral MD-TN (voxel threshold *p* < 0.05). **(B)**. Significantly reduced seed-based FC from the MD-TN in AUD with a voxel-level threshold of *p* < 0.01. **(C)** Significantly reduced seed-based FC from the MD-TN in AUD with a voxel-level threshold of *p* < 0.05 (for visualization purposes, see Supplementary Material S1).

**Figure 3:**
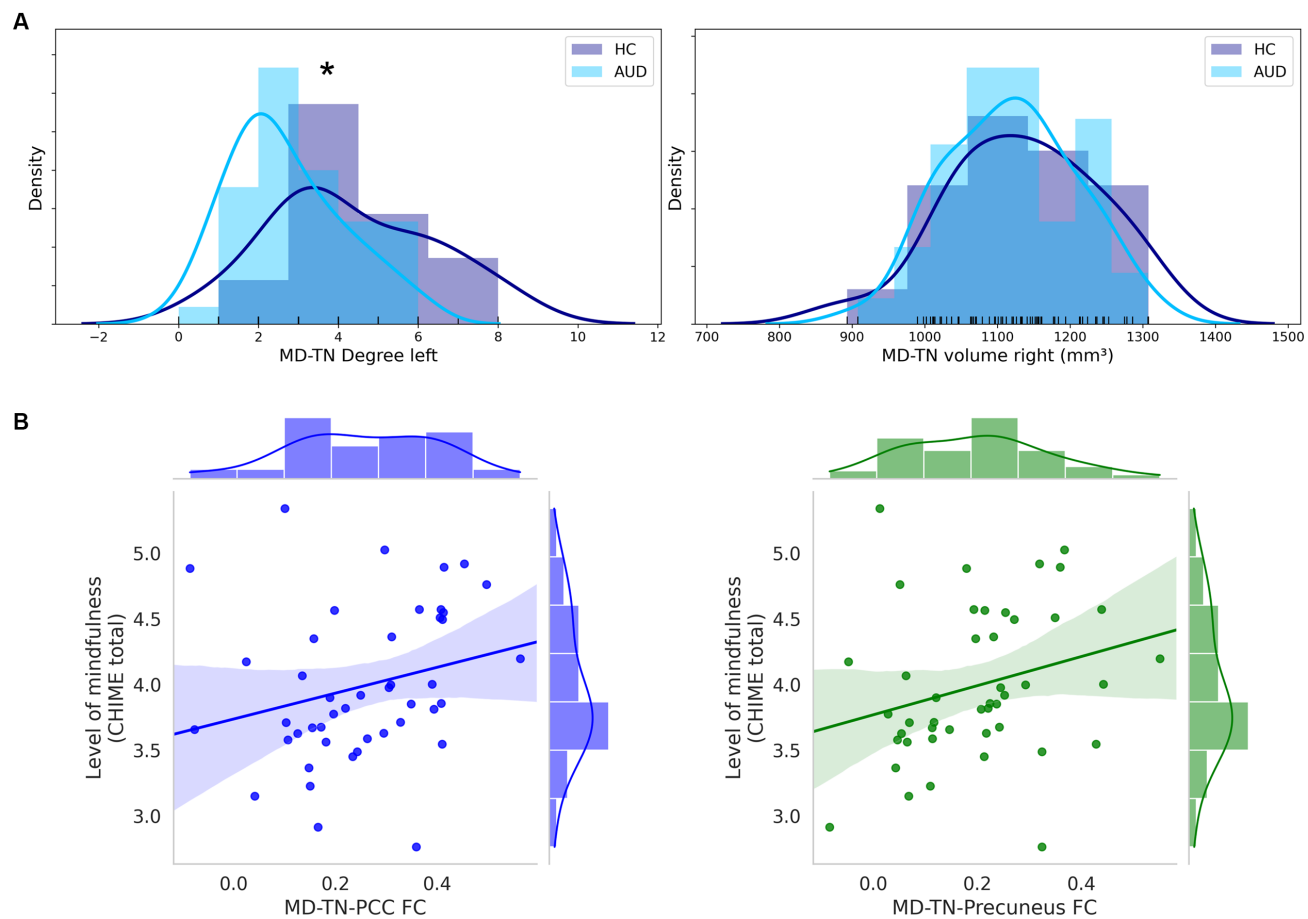
MD-TN network centrality and significant correlations of MD-TN functional connectivity with mindfulness. **(A)** Histograms of group distributions of left and right MD-TN network centrality degree. **(B)** Significant positive correlation of the MD-TN-PCC and MD-TN-precuneus FC and levels of mindfulness within AUD patients. *Significant ANVOCA *p*-value.

In comparison to HC, patients with AUD had significantly lower MD-TN associated FC in two large clusters, including the ACC (2001 voxel) and the insular cortex (957 voxel) (see Table 3, Figure 2). In comparison to HC, patients showed significantly lower network centrality degree of the left MD-TN [2.69 ± 1.44 vs. 4.35 ± 2.06 mm^3^, *F*(1, 60) = 14.38, *p* < 0.001, large effect size *η*^2^ = 0.193] (see Figure 3A). No significant group difference between patients and HC was found for the right MD-TN [3.04 ± 1.62 vs. 3.05 ± 2.37 mm^3^, *F*(1, 60) = 0.071, *p* < 0.790]. See Supplementary Table S1 for results with a voxel-level of *p* < 0.05.

**Table 3:** Whole-brain seed-based FC of the MD-TN (voxel-level *p* < 0.01).

Contrast	Size (voxels)	Peak (MNI)	*T*-value	*p*-FWE
*HC > AUD patients*				
Cingulate Gyrus, anterior division	2001	-36 -12 -06	4.00	<0.001
Insular cortex left	957	0 4 30	5.14	0.023
*AUD patients > HC*				
None	n/a	n/a	n/a	n/a

n/a, not applicable.

### 3.5. Exploratory correlations

Within all subjects only employment correlated significantly with levels of mindfulness (years of education: *r* = 0.143, *p* = 0.260; employment: *r* = 0.254, *p* = 0.041; relationship: *r* = 0.123, *p* = 0.329). No significant correlations were found in the HC (years of education: *r* = 0.368, *p* = 0.121; employment: *r* = 0.219, *p* = 0.354; relationship: *r* = −0.085, *p* = 0.722) and AUD (years of education: *r* = −0.045, *p* = 0.769; employment: *r* = 0.189, *p* = 0.213; relationship: *r* = 0.068, *p* = 0.655).

In both patients and HC, there were no significant correlations between levels of mindfulness (CHIME total) and the volumetric measures. However, within patients with AUD there was a positive trend for a correlation between CHIME total and volumes of the left (*r* = 0.242, *p* = 0.109) and right (*r* = 0.254, *p* = 0.092) MD-TN. Within patients, levels of mindfulness (CHIME total) correlated positively with the MD-TN-PCC FC (*r* = 0.362, *p* = 0.015) and MD-TN-precuneus FC (*r* = 0.305, *p* = 0.042) (see Figure 3B). No correlation was found for the MD-TN-ACC FC (*r* = 0.051, *p* = 0.890). Within HC, no significant correlation was found between level of mindfulness and FC values (MD-TN-ACC: *r* = 0.068, *p* = 0.777; MD-TN-PCC: *r* = −0.102, *p* = 0.668; MD-TN-precuneus: *r* = 0.117, *p* = 0.662).

Exploratory correlations of days of abstinence revealed a positive correlation with CHIME subscore acceptance (*r* = 0.431, *p* = 0.004) and a negative correlation with CHIME subscore openness (*r* = −0.307, *p* = 0.045). Number of detoxifications correlated negatively with CHIME subscore outer awareness (*r* = −0.509, *p* = 0.013) (see Supplementary Figure S1A). Days of abstinence correlated positively with the left (*r* = 0.329, *p* = 0.031) and right MD-TN volume (*r* = 0.389, *p* = 0.010), and with the left (*r* = 0.327, *p* = 0.032) and right network centrality degree of the MD-TN (*r* = 0.479, *p* = 0.001) (see Supplementary Figure S1B). Within AUD CHIME’s subscores inner and outer awareness correlated positively with MD-TN-PCC FC (*r* = 0.421, *p* = 0.004; *r* = 0.411, *p* = 0.005) and MD-TN-precuneus FC (*r* = 0.357, *p* = 0.016; *r* = 0.291, *p* = 0.052, trend) in AUD. CHIME’s subscores acceptance and decentering correlated positively with the left (*r* = 0.248, *p* = 0.100, trend; *r* = 0.347, *p* = 0.020) and right MD-TN volume (*r* = 0.302, *p* = 0.044; *r* = 0.344, *p* = 0.021). CHIME’s subscore relativity correlated negatively with cortical thickness of the right hemisphere (*r* = −0.297, *r* = 0.047). CHIME’s subscores insight correlated positively with volume of the left (*r* = 0.347, *p* = 0.020) and right MD-TN volume (*r* = 0.329, *p* = 0.027), and negatively with cortical thickness of the right hemisphere (*r* = −0.332, *p* = 0.026). CHIME’s subscore openness correlated negatively with network centrality degree of the right MD-TN (*r* = 0.383, *p* = 0.009) (see Supplementary Figure S1C).

## 4. Discussion

This is the first multimodal MRI study investigating the association between the neurobiology of the thalamocortical system and mindfulness in AUD. Levels of mindfulness were reduced in patients with AUD. Results were driven by the subscores acceptance, decentering and openness, pointing to a reduction of specific aspects of mindfulness in patients with AUD. We identified lower bilateral thalamic and left MD-TN volumes in patients as compared to HC. Analysis of resting-state fMRI revealed lower FC of the MD-TN to the SN (ACC, insula) in patients with AUD. In addition, we found lower left MD-TN network centrality degree in AUD, suggesting reduced functional integration of the MD-TN in comparison to HC. AUD patients with lower scores of mindfulness showed lower FC of the MD-TN-PCC and MD-TN-precuneus.

We identified lower levels of mindfulness in patients with AUD. This is of clinical importance because previous studies in patients showed a direct link between lower levels of mindfulness and impulsivity traits (11), with increased craving (21) and reduced abstinence-related self-efficacy (58). These associations are important in understanding the effectiveness of mindfulness-based interventions in general and specific aspects of mindfulness in patients with AUD, such as mindfulness-based relapse prevention (MBRP) (59, 60) or mindfulness-based addiction treatment (MBAT) (61), for reducing alcohol use and craving (59–62).

On a neurobiological level mindfulness is associated with alterations of the thalamocortical system. We identified lower bilateral thalamic and left MD-TN volume in patients as compared to HC, which is in line with findings of previous studies, including a large multicentre study from the ENIGMA Addiction Working Group (63–65). A novel aspect of our structural analyses is that we investigated the association between volumes and mindfulness. Exploratory correlations suggest that lower volumes of the MD-TN are associated with impaired decentering, insight and acceptance in patients. Interestingly, MD-TN volumes were higher within patients with longer abstinence (see Supplementary Materials S2, S3). This is in-line with the observation that continuing alcohol abstinence in AUD results in an increase of subcortical volumes (66).

Furthermore, we found reduced FC between the MD-TN and the SN (ACC, insula) and by investigating *a priori* regions with the DMN (precuneus, PCC) in patients with AUD. These results are in line with previous findings of reduced thalamic FC to midline regions including the ACC (67). Reductions in thalamocortical FC may decrease levels of mindfulness or vice versa, hereby deteriorating the likelihood of maintaining abstinence. Our results propose that in patients with AUD, communication of the MD-TN is perturbated. With the MD-TNs role as an integrator and selector of mental representations, this may be relevant for mindful procession of thoughts and actions. The DMN is known to play a central role for mindfulness, and both the precuneus and PCC have been implicated as a neural substrate of consciousness and awareness, as shown by studies in disorders of consciousness (68). This is supported by our data demonstrating that MD-TN’s FC to the posterior DMN (precuneus and PCC) is positively associated with levels of awareness (CHIME: inner and outer awareness) in AUD.

Methodologically, our analyses extend prior studies in several ways. First, we specifically investigated the MD-TN, a thalamic subregion with particular importance for communication with the DMN and the SN. Second, we complement our analyses using a graph theory approach. We identified lower overall network centrality degree of the left MD-TN, allowing conclusions of reduced whole brain communication of the MD-TN in patients with AUD as compared to HC. Matching those findings of impaired communication and integration of the MD-TN, Hade et al. (69) found molecular genetic changes in the MD-TN in patients with AUD, which indicates a development of AUD-related dysregulation of neurocircuitry. These alterations may be caused by the neurotoxic effect of alcohol, which is supported by our finding of lower MD-TN volumes in patients with shorter duration of abstinence (70).

Taken together, our analyses revealed structural and functional alterations of the MD-TN associated thalamocortical system in patients. It is possible that reduced FC is related to structural disturbances in neurocircuitry of the MD-TN in AUD (69). These alterations may be caused by the neurotoxic effect of alcohol, which is supported by our finding of lower MD-TN volumes in patients with shorter duration of abstinence (70).

Our study has some limitations. First, based on 3 Tesla MRI data it is not possible to visually delineate the MD-TN. This may result in resolution-based limitations of volume measurements. However, our finding is in line with findings derived from diffusion tensor imaging (DTI) based parcellation of thalamic nuclei (71). High-field acquisition using 7-Tesla MRI may be helpful to further increase precision of delineation and volume measurement of thalamic nuclei (72, 73). Second, our assessment of mindfulness cannot discriminate between personality like components of mindfulness and the lasting impact of other factors, e.g., AUD or psychotherapeutic interventions on mindfulness. A meta-analytic investigation showed that factors of mindfulness are not invariable traits and could be altered over time and through mindfulness training (17). It is noteworthy that we found greatest differences between AUD and HC participants in mindfulness factors of acceptance, decentring and openness, whereas within the AUD group additional mindfulness factors (inner/outer awareness and insight) seems to be associated with MD-TN characteristics. Further investigations of longitudinal mindfulness measurements may contribute to understand, which mindfulness-related factors can be improved by means of continuous alcohol abstinence or through clinical interventions. Third, our correlational analyses are exploratory and not controlled for multiple comparisons. This approach seems justified because this is the first study that uses multimodal MRI to investigate distinct features of mindfulness in AUD. However, we acknowledge that future replication studies are needed to confirm our correlational findings. Fourth, our sample size was quite small and larger studies are needed to confirm findings in mindfulness and MD-TN characteristics. Our groups differ significantly for years of education and binary variables employment and relationship, which may have an impact on levels of mindfulness. MRI findings were controlled for years of education. For our exploratory correlations with levels of mindfulness, we did not control for those variables. However, within AUD and HC these variables did not correlate significantly with levels of mindfulness.

In conclusion, our findings point to reduced levels of mindfulness in patients with AUD and AUD related alterations in areas of the MD-TN important for propagating mindfulness. Specifically, we found reduced volume, integration and communication of the MD-TN in AUD. Exploratory correlations suggests an association of impaired structural and functional MD-TN measurements with lower values of mindfulness in AUD. The role of the MD-TN extends prior knowledge of addiction-specific, limbic, and orbitofrontal circuits. Our findings contribute to understand the importance of mindfulness and the thalamocortical network in AUD.

## Data availability statement

The raw data supporting the conclusions of this article will be made available by the authors, without undue reservation.

## Ethics statement

The studies involving human participants were reviewed and approved by cantonal ethics committee of Bern (KEK Bern; KEK-number: 2016-00988). The patients/participants provided their written informed consent to participate in this study.

## Author contributions

ND, LS, MS, FM, and TB conceived the idea and methodology of the study. ND analyzed the MRI data, conducted the statistical analyses and wrote the manuscript. LS, MS, and MG recruited subjects and were involved in clinical and diagnostic assessments and managed the MRI and clinical data. AF and RW provided technical support for MRI scanning and data processing. ZK provided support for mindfulness assessments. All authors contributed to the article and approved the submitted version.

## Funding

The study was funded by a grant from the Swiss Foundation for Alcohol Research (SSA-no. 283), by the Swiss National Science Foundation (SNF no. 105319_159286) and by the Novartis-Foundation for Medical-Biological Research (no. 19A063). Tobias Bracht received a scholarship from the Adrian et Simone Frutiger Foundation.

## Conflict of interest

The authors declare that the research was conducted in the absence of any commercial or financial relationships that could be construed as a potential conflict of interest.

## Publisher’s note

All claims expressed in this article are solely those of the authors and do not necessarily represent those of their affiliated organizations, or those of the publisher, the editors and the reviewers. Any product that may be evaluated in this article, or claim that may be made by its manufacturer, is not guaranteed or endorsed by the publisher.
