# Supplementary material for: Associations of thalamocortical networks with reduced mindfulness in alcohol use disorder
Source: Front Psychiatry. 2023 Jul 6;14:1123204. doi: 10.3389/fpsyt.2023.1123204 (PMC10358776; doi:10.3389/fpsyt.2023.1123204)
Supplement: Supplementary file 1 [file Data_Sheet_1.docx]

Supplementary Material

Associations of thalamocortical networks with reduced mindfulness in alcohol use disorder

Niklaus Denier*, Leila M. Soravia, Franz Moggi, Maria Stein, Matthias Grieder, Andrea Federspiel, Zeno Kupper, Roland Wiest, Tobias Bracht

*** Correspondence:** Niklaus Denier: [niklaus.denier@upd.unibe.ch](mailto:niklaus.denier@upd.unibe.ch)

# Supplementary Figures and Tables

## Supplementary Tables

**Supplementary Table 1:** Whole-brain seed-based FC of the MD-TN (voxel-level: p < 0.05).

| **Contrast** | **Size (voxels)** | **Peak (MNI)** | **T-value** | **p-FWE** |
| --- | --- | --- | --- | --- |
| **HC > AUD patients** |  |  |  |  |
| Midline area | 4485 | 0 4 30 | 5.14 | 0.007 |
| Cingulate Gyrus, anterior division | 1409 |  |  |  |
| Cingulate Gyrus, posterior division | 646 |  |  |  |
| Precuneous Cortex | 447 |  |  |  |
| Frontal Pole Right | 203 |  |  |  |
| Paracingulate Gyrus Left | 135 |  |  |  |
| Temporo-frontal lobe left | 8339 | -36 -12 -6 | 4.00 | <0.001 |
| Insular Cortex | 708 |  |  |  |
| Frontal Pole | 641 |  |  |  |
| Supramarginal Gyrus, anterior division | 595 |  |  |  |
| Central Opercular Cortex | 556 |  |  |  |
| Parietal Operculum Cortex | 438 |  |  |  |
| Planum Temporale | 377 |  |  |  |
| Middle Frontal Gyrus | 333 |  |  |  |
| Putamen | 277 |  |  |  |
| Heschl's Gyrus | 270 |  |  |  |
| Inferior Frontal Gyrus, pars opercularis | 257 |  |  |  |
| Middle Temporal Gyrus, posterior division | 213 |  |  |  |
| Postcentral Gyrus | 200 |  |  |  |
| Inferior Frontal Gyrus, pars triangularis | 199 |  |  |  |
| Supramarginal Gyrus, posterior division | 148 |  |  |  |
| Precentral Gyrus | 137 |  |  |  |
| Planum Polare | 136 |  |  |  |
| Superior Temporal Gyrus, posterior division | 103 |  |  |  |
| Temporo-frontal lobe right | 4022 | 36 -32 20 | 3.61 | 0.014 |
| Insular Cortex | 567 |  |  |  |
| Middle Temporal Gyrus, posterior division | 416 |  |  |  |
| Parietal Operculum Cortex | 341 |  |  |  |
| Central Opercular Cortex | 266 |  |  |  |
| Heschl's Gyrus | 264 |  |  |  |
| Inferior Temporal Gyrus, posterior division | 154 |  |  |  |
| Supramarginal Gyrus, posterior division | 138 |  |  |  |
| Planum Temporale | 128 |  |  |  |
| Planum Polare | 127 |  |  |  |
| Temporal Pole | 120 |  |  |  |
| Frontal Orbital Cortex | 104 |  |  |  |
| **AUD patients > HC** |  |  |  |  |
| None | n/a | n/a | n/a | n/a |

Only regions with more than 100 voxels are listened. n/a: not applicable.

## Supplementary Figures

**Supplementary figure 1:** Exploratory correlation analyses between CHIME subscores and alcohol related scored and neuroimaging findings within AUD patients.


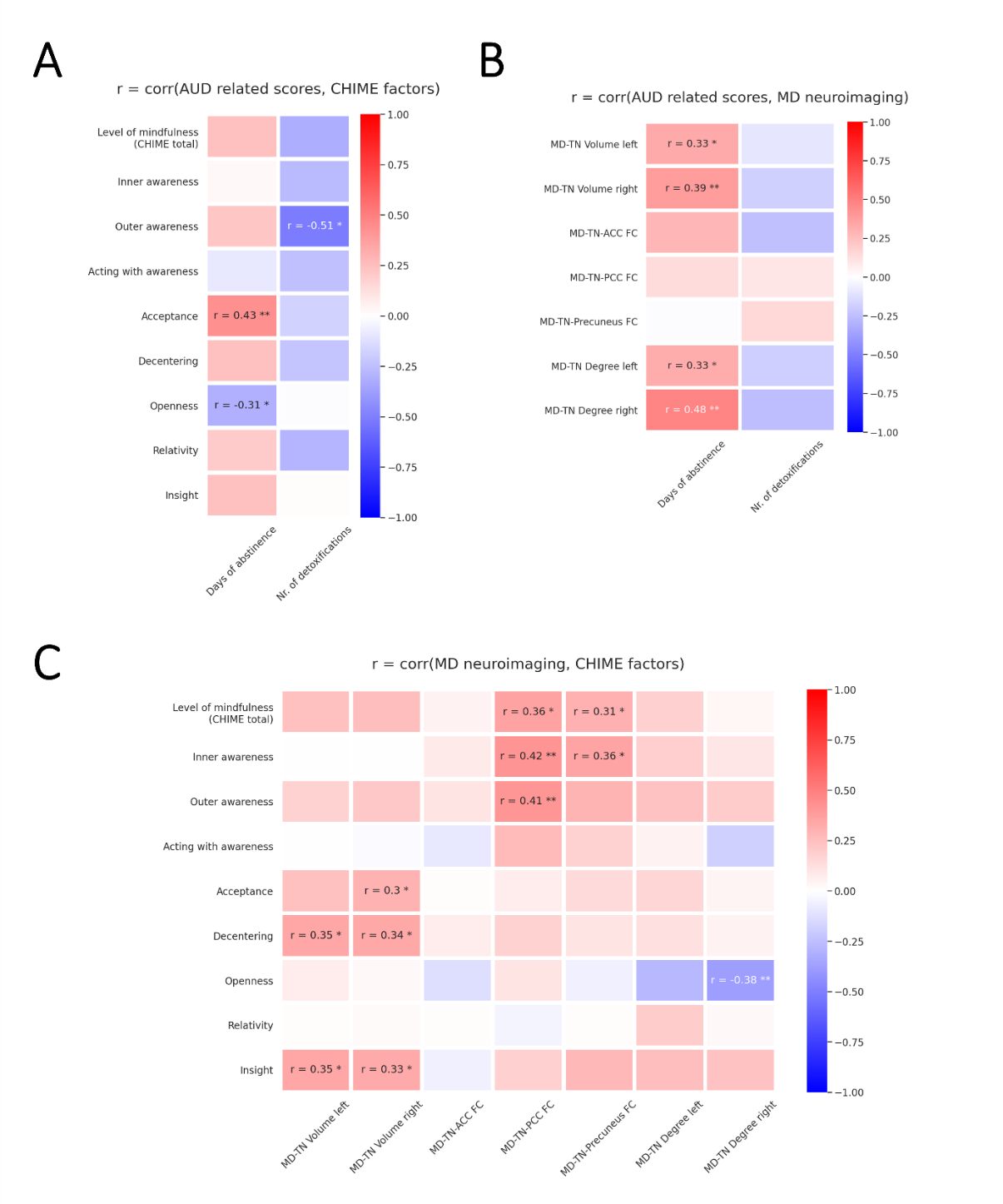


Heat maps with colours ranging from blue to red represent correlation coefficients (-1 … 1), values are only showed for significant correlations (*: p < 0.05; **: p < 0.01). **A)** Correlation heat map of CHIME subscores and AUD related scores. **B)** Correlation heat map of AUD related scores and structural and functional aspects of the MD-TN. **C)** Correlation heat map of CHIME subscores and structural and functional aspects of the MD-TN.
